# Supplementary material for: Content-rich biological network constructed by mining PubMed abstracts
Source: BMC Bioinformatics. 2004 Oct 8;5:147. doi: 10.1186/1471-2105-5-147 (PMC528731; doi:10.1186/1471-2105-5-147)
Supplement: Additional File 2 — The original results of the above study (non-essential files are deleted to keep the file size under the limit set by BMC bioinformatics). [file 1471-2105-5-147-S2.bz2 › chilibotAdditionalFile2/dip05/107ID1311321E442/html/MYOG_TCF3.html]

 


 **MYOG** and **TCF3** 
  
Found 23 abstracts in PubMed, retrieved 05.  
 

 What does Google say? 
 PDF only 
| .edu only 

---

**Interactive relationship** (e.g. stimulation, inhibition, etc)

**Neutral relationship**- Here, we report that Pbx Meis1 Prep1 binds DNA cooperatively with heterodimers of E2a  [ **TCF3** ]  and MyoD, myogenin  [ **MYOG** ] , Mrf 4 or Myf 5.  Ref: 10471746 Nucleic Acids Res, 1999

**Non-interactive relationship** (e.g. studied together, co-existance, homology, etc.)

- Moreover, while ITF  [ **TCF3** ]  2b inhibited the creatine kinase promoter, it acted as a weak transactivator on an artificial promoter consisting of three tandem copies of the consensus myogenic factor  [ **MYOG** ]  DNA binding site.  Ref: 8999959 J Biol Chem, 1997
- The myogenic regulatory factors MRFs, MyoD, myogenin  [ **MYOG** ] , Myf 5, and MRF4, function as transcriptional activators of muscle specific gene expression by forming heterodimers with the ubiquitously expressed products of the E2A  [ **TCF3** ]  gene, E12 and E47.  Ref: 9116507 Protein Expr Purif, 1997
